# The Impact of HIV Infection and CD4 Cell Count on the Performance of an Interferon Gamma Release Assay in Patients with Pulmonary Tuberculosis

**DOI:** 10.1371/journal.pone.0004220

**Published:** 2009-01-19

**Authors:** Martine G. Aabye, Pernille Ravn, George PrayGod, Kidola Jeremiah, Apolinary Mugomela, Maria Jepsen, Daniel Faurholt, Nyagosya Range, Henrik Friis, John Changalucha, Aase B. Andersen

**Affiliations:** 1 Department of Infectious Diseases, University of Copenhagen, Rigshospitalet, Copenhagen, Denmark; 2 Unit for Infectious Diseases Q, University of Copenhagen, Herlev Hospital, Herlev, Denmark; 3 National Institute for Medical Research, Mwanza Medical Research Centre, Mwanza, Tanzania; 4 Zonal Tuberculosis Reference Laboratory, Bugando Medical Centre, Mwanza, Tanzania; 5 Department of Human Nutrition, Faculty of Life Sciences, University of Copenhagen, Frederiksberg, Denmark; 6 National Institute for Medical Research, Muhimbili Medical Research Centre, Dar Es Salaam, Tanzania; University of Stellenbosch, South Africa

## Abstract

**Background:**

The performance of the tuberculosis specific Interferon Gamma Release Assays (IGRAs) has not been sufficiently documented in tuberculosis- and HIV-endemic settings. This study evaluated the sensitivity of the QuantiFERON TB-Gold In-Tube (QFT-IT) in patients with culture confirmed pulmonary tuberculosis (PTB) in a TB- and HIV-endemic population and the effect of HIV-infection and CD4 cell count on test performance.

**Methodology/Principal Findings:**

161 patients with sputum culture confirmed PTB were subjected to HIV- and QFT-IT testing and measurement of CD4 cell count. The QFT-IT was positive in 74% (119/161; 95% CI: 67–81%). Sensitivity was higher in HIV-negative (75/93) than in HIV-positive (44/68) patients (81% vs. 65%, p = 0.02) and increased with CD4 cell count in HIV-positive patients (test for trend p = 0.03). 23 patients (14%) had an indeterminate result and this proportion decreased with increasing CD4 cell count in HIV-positive patients (test for trend p = 0.03). Low CD4 cell count (<300 cells/µl) did not account for all QFT-IT indeterminate nor all negative results. Sensitivity when excluding indeterminate results was 86% (95% CI: 81–92%) and did not differ between HIV-negative and HIV–positive patients (88 vs. 83%, p = 0.39).

**Conclusions/Significance:**

Sensitivity of the QFT-IT for diagnosing active PTB infection was reasonable when excluding indeterminate results and in HIV-negative patients. However, since the test missed more than 10% of patients, its potential as a rule-out test for active TB disease is limited. Furthermore, test performance is impaired by low CD4 cell count in HIV-positive patients and possibly by other factors as well in both HIV-positive and HIV-negative patients. This might limit the potential of the test in populations where HIV-infection is prevalent.

## Introduction

There is a great demand throughout the world for new methods for diagnosing both active and latent tuberculosis infection. New methods for indirect tuberculosis (TB) diagnosis are now available, including the Interferon Gamma Release Assays (IGRAs). IGRAs are T-cell-based assays relying on the principle that sensitised T-cells from a whole blood sample produce the cytokine Interferon-gamma (IFN-γ) when incubated with antigens specific for *M.tuberculosis* (ESAT-6, CFP-10, TB-Antigen 7.7).

There are several potential uses of these tests. As the tests do not distinguish between active and latent TB infection, their potential for the diagnosis of active TB infection in TB endemic areas is limited [1]. However, in evaluation of IGRA performance active TB infection has commonly been used as a surrogate marker for LTBI, while other studies have employed risk of exposure. Recent prospective studies have shown that IGRAs might be more accurate and thus have a higher predictive value in the diagnosis of LTBI than does the conventional tuberculin skin test (TST) and a role of IGRAs in ruling out TB disease in patients suspected of TB has also been proposed [2]–[5]. The tests have already been taken into use in clinical practice in several countries with low prevalence of latent TB infection (LTBI).

HIV-infection increases the risk of progression from LTBI to active TB disease and preventive treatment of LTBI in HIV-positive individuals is increasingly recommended necessitating an efficient test for diagnosing LTBI [6]. Although reports have shown that IGRAs have a very impressive specificity for TB infection, no golden standard is available for the diagnosis of LTBI which makes sensitivity estimation difficult [7].

Two IGRAs are currently commercially available: the ELISA-based QuantiFERON-TB® Gold In tube test (QFT-IT; Cellestis Limited, Australia) and the EliSPOT-based T-SPOT.*TB*® test (Oxford Immunotec, Abingdon, United Kingdom). The QFT-IT has recently received approval from the U.S. Food and Drugs Administration [8] and recent guidelines are now recommending the use of IGRAs for diagnosing LTBI as an alternative to the TST [7], [9]. A recent review reported a pooled sensitivity of 70% for the QFT-IT (95% CI: 65–78%) and 90% for the T-SPOT.*TB* (95% CI: 86–93%)[9]. However, published studies in this area are highly variable in respect to sample size, study population etc. The majority of studies of the QFT-IT have been carried out in high-income settings and not in TB-and HIV-endemic countries where the burden is highest. Since IGRAs rely on immune response, their performance may be impaired in immunocompromised populations and in order to determine the applicability of IGRAs in these areas, more studies from such populations are needed. Previous studies in HIV-infected populations have generally concluded that the QFT-IT is less influenced by immune anergy than is the TST, but results are diverging [5], [7], [10]–[16]. To our knowledge, only two studies have been published on the performance of the QFT-IT in populations and under conditions similar to ours. Raby et al [16] found an overall sensitivity of the QFT-IT of 74% in patients with smear positive PTB with lower sensitivity in 59 HIV-positive compared to 96 HIV-negative patients (84 vs. 63%, p = 0.03). This study also reported a decrease in sensitivity with decreasing CD4 cell count in patients with a CD4 cell count below 350 cells/µl as well as an association between low CD4 cell count and a negative and indeterminate QFT-IT test result. Tsiouris et al [14] found an overall sensitivity of 76% and, though not significantly so, similarly found a lower sensitivity in 26 HIV-positive patients compared to 15 HIV-negative patients with culture confirmed TB (65 vs. 73%, Fisher's exact test p = 0.73; calculated by authors from crude data in article). They also found a significantly lower mean level of IFN-γ in HIV-positive patients compared to HIV-negative patients (1.3 vs. 8.2 IU/ml, p = 0.03). Studies have reported similar results for the T-SPOT.*TB* test [17], [18], while others have reported that the T-SPOT.*TB* might be less affected by immunodeficiency than is the QFT-IT [10], [15], [19].

The present study evaluated the sensitivity of the QFT-IT in patients with culture confirmed pulmonary TB disease (PTB) in a TB- and HIV-endemic adult patient population in Tanzania and evaluated the influence of HIV-status and CD4 cell count on test performance.

## Results

### Patient characteristics

161 patients were included in the study. Demographic and clinical characteristics of study participants are summarized in **Table 1**. More men than women participated in the study (104 vs. 57, p<0.01) and the male to female rate was 1.82∶1. A total of 68 patients (42%) were HIV-positive. Compared to men, a higher proportion of women were HIV-positive (35 vs. 56%, p = 0.01). Median lymphocyte count was 1626 cells/µl (inter-quartile range 1243–2224 cells/ µl) and median CD4 cell count was 377 cells/µl (inter-quartile range 249–665 cells/µl).

**Table 1:** Demographic and clinical characteristics of patients according to HIV status.

Category	Subcategory	HIV-positive	HIV-negative	p-value*
		(n = 93)	(n = 68)	
Male sex, n (%)		68 (73.1)	36 (52.9)	0.01
Age, median (IQR)		28 (23–37)	35 (29–42)	<0.01
BMI, median (range)		18.3 (13.2–23.8)	17.7 (12.0–27.6)	0.03
Lymphocyte count/µl, median (IQR)		1640 (1252–2263)	1510 (1230–2218)	0.80
CD4 cell count/µl, median (IQR)		519 (317–778)	272 (172–478)	<0.01
Smear grade†	0, n (%)	18 (19)	17 (25)	0.39
	1–9, n (%)	0 (0)	0 (0)	n/a
	1+, n (%)	6 (6)	11 (16)	0.05
	2+, n (%)	22 (24)	11 (16)	0.25
	3+, n (%)	42 (45)	28 (41)	0.61
	Unknown, n (%)	5 (6)	1 (2)	

* p-value for difference between HIV-negative and HIV–positive patients. IQR: Inter-quartile range.

† Smear grade by sputum microscopy: 0 (no acid fast bacilli [AFB] on smear), 1–9 (exact number of AFB per 100 fields), 1+ (10–99 AFB per 100 fields), 2+ (1–10 AFB per field) and 3+ (more than 10 AFB per field).

### QFT-IT results and sensitivity

Of the 161 patients included, 119 (74%, 95% CI: 67–81%) had a positive, 19 (12%, 95% CI: 7–17%) had a negative and 23 (14%, 95% CI: 9–20%) had an indeterminate QFT-IT result (**Table 2**). All 23 indeterminate results were due to an insufficient response to both the PHA and the *M.tb.*-specific antigens. When excluding indeterminate results sensitivity was 86% (95% CI: 81–92%).

**Table 2:** QuantiFERON-TB® Gold In-tube results for all study participants and according to HIV status.

QFT-IT result	All	HIV-negative	HIV-positive	p-value*
	(n = 161)	(n = 93)	(n = 63)	
Positive, n (%)	119 (74)	75 (81)	44 (65)	0.02
Negative, n (%)	19 (12)	10 (11)	9 (13)	0.63
Indeterminate, n (%)	23 (14)	8 (9)	15 (22)	0.02

QFT-IT: QuantiFERON-TB® Gold In-tube test.

* p-value for difference between HIV-negative and HIV–positive patients.

Odds ratios for possible explanatory parameters for an indeterminate QFT-IT result are listed in **table 3**. The following parameters were not found to be associated with an indeterminate QFT-IT result: body mass index (BMI), lymphocyte count above the median of 1640 cells/µl, sputum microscopy smear grade of 3+. Factors associated with an indeterminate result were: CD4 cell count below 300 cells/µl (by both univariate and multivariate analysis), age above 33 years (by univariate analysis only) and male sex (by multivariate analysis only). None of the mentioned parameters were associated with a negative QFT-IT result and no other differences were found when comparing medians and proportions of these parameters between patients with positive, negative and indeterminate results respectively.

**Table 3:** Association of risk factors with an indeterminate QuantiFERON-TB® Gold In-tube result.

Parameter	n	Univariate analysis		Multivariate analysis	
		OR (95% CI)	p	OR (95% CI)	p
Male sex	104	2.96 (0.96–9.18)	0.06	3.25 (1.00–10.49)	0.05
Age>33 years	71	2.75 (1.09–6.91)	0.03	2.51 (0.56–11.28)	0.23
BMI <18.5	98	0.81 (0.33–1.98)	0.64	0.68 (0.26–1.77)	0.43
CD4 cell count <300 cells/µl	62	2.83 (1.14–7.00)	0.02	3.41 (1.25–9.29)	0.02
Lymphocyte count <1640 cells/µl	83	1.42 (0.58–3.45)	0.44	1.21 (0.43–2.92)	0.81
3+ AFB by sputum smear microscopy	70	1.86 (0.61–5.67)	0.27	1.57 (0.57–4.27)	0.38

A total of 23 out of 161 patients (14%) had a QFT-IT indeterminate result. No differences were found in OR when comparing QFT-IT positive and negative patients. OR: Odds Ratio. CI: Confidence interval. BMI: Body Mass Index. Age of 33 years and lymphocyte count of 1640 cells/µl represents medians for all patients. 3+ AFB by sputum smear microscopy: More than 10 acid fast bacilli (AFB) per field.

### Impact of HIV-status and CD4 cell count

BMI was significantly lower in HIV-positive than HIV–negative patients (18.0 vs. 18.5, p = 0.03) as was median CD4 cell count (272 vs. 519 cells/µl, p<0.01) (table 1).

**Figure 1:**
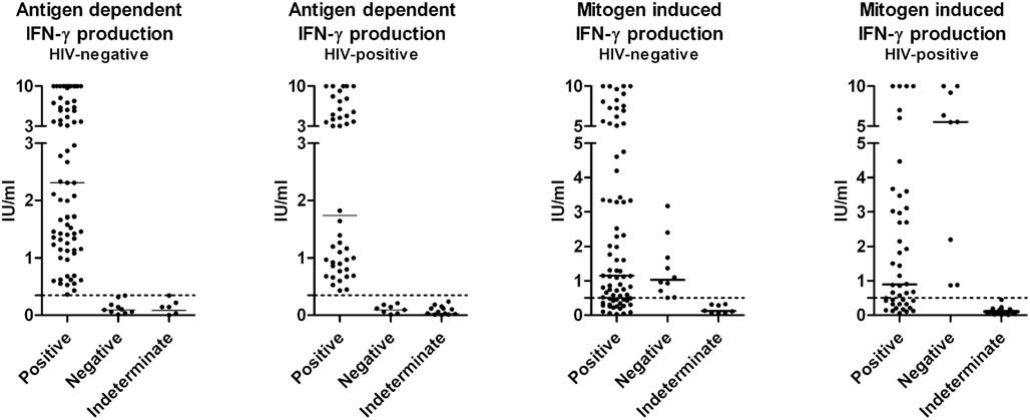
Antigen dependent and mitogen induced absolute IFN-γ levels by QuantiFERON-TB® Gold In-tube test result in HIV-negative and HIV-positive patients respectively. Horisontal lines represent medians. Dotted lines represent the applied cut-off values as recommended by the manufacturer: 0.35 IU/ml for antigen (ESAT-6, CFP-10, TB7.7) dependent IFN-γ production and 0.50 IU/ml for mitogen (PHA) induced IFN-γ production respectively. The assay is not able to quantify values above 10 IU/ml why values above this limit were assigned the value 10 IU/ml. No significant differences in median levels between HIV-positive and HIV-negative were observed.

**Figure 2:**
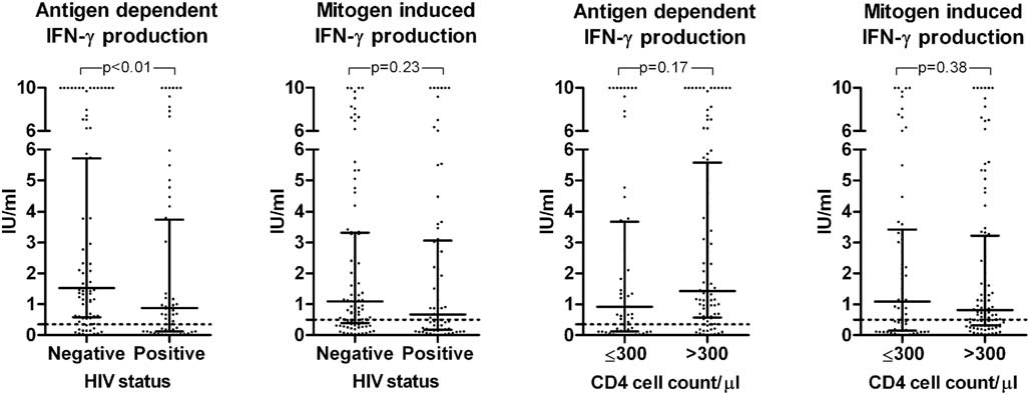
Antigen dependent and mitogen induced absolute IFN-γ levels by HIV-status and CD4 cell count group. Horisontal lines represent medians with interquartile range. Dotted lines represent the applied cut-off values as recommended by the manufacturer: 0.35 IU/ml for antigen (ESAT-6, CFP-10, TB7.7) dependent IFN-γ production and 0.50 IU/ml for mitogen (PHA) induced IFN-γ production respectively. The assay is not able to quantify values above 10 IU/ml why values above this limit were assigned the value 10 IU/ml.

Median antigen dependent and mitogen induced IFN-γ production for QFT-IT positive, negative and indeterminate results for HIV-negative and HIV-positive patients respectively are depicted in **figure 1**. No significant differences were found in levels of IFN-γ between HIV-positive and HIV-negative patients when looking at each group of test results (p>0.05). However, as shown in **figure 2**, overall median values of antigen dependent IFN-γ production were lower in HIV-positive than in HIV-negative patients (p = 0.02). This was not the case for the mitogen induced IFN-γ production. No significant differences in IFN-γ were found between patients with a CD4 cell count above compared to below 300 cells/µl.

Sensitivity of the QFT-IT was lower in HIV-positive than in HIV–negative patients (65 [95% CI: 53–76%] vs. 81% [95% CI: 73–89%], p = 0.02) which was due to a larger proportion of QFT-IT indeterminate results among HIV-positive patients compared to HIV-negative patients (22 vs. 9%, p = 0.02). When excluding indeterminate results, sensitivity increased to 83% in HIV-positive (44/53; 95% CI: 73–93%) and to 88% (75/85; 95% CI: 79–94%) in HIV-negative patients and no difference was observed in sensitivity between the HIV-positive and HIV–negative patients (p = 0.39). Furthermore, when excluding QFT-IT indeterminate results no significant difference was found in median antigen dependent IFN-γ production (p = 0.20).

**Figure 3:**
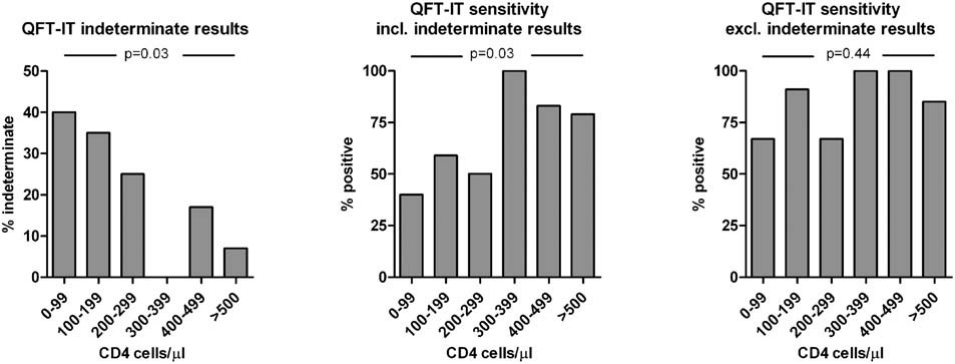
Influence of CD4 cell count on performance of the QuantiFERON-TB® Gold In-tube test in HIV-positive patients. For HIV-positive patients the % of indeterminate and positive test responders respectively was grouped by the individual number of CD4 cells/µl. P-values are for Cochrane-Armitage test for trend. A similar relationship was not found in HIV-negative patients. The number of patients in each CD4 cell group was: 0–99: 5, 100–199: 17, 200–299: 20, 300–399∶6, 400–499∶6, >500∶14. QFT-IT: QuantiFERON-TB® Gold In-tube test.

A positive trend of sensitivity with increasing CD4 cell count was observed for HIV-positive patients when stratifying CD4 cell counts into groups of <100, 100–200, 200–300, 300–400, 400–500, >500 (p = 0.03). We found a similar inverse trend with increasing proportion of indeterminate results with decreasing CD4 cell count (p = 0.03). When excluding indeterminate results no trend was observed for sensitivity and CD4 cell count (p = 0.44) (**Figure 3**). These findings were observed both when dividing CD4 cell counts into groups of pentiles and when dividing them into groups of hundreds. In HIV-positive patients, QFT-IT sensitivity was significantly lower in patients with CD4 cell count below compared to above 300 cells/µl (52 vs. 85%, p = 0.01). This was not the case for HIV-negative patients (90 vs. 78%, p = 0.35).

## Discussion

This study evaluated the performance of the QFT-IT test in a TB- and HIV-endemic population. A sputum culture positive for *M.tb.* was used as standard for present TB infection and the influence of HIV-infection and CD4 cell count on test performance was investigated as were risk factors for a negative or indeterminate QFT-IT result.

The observed sensitivity of 74% when including indeterminate results is in the lower end of the spectre for previously reported studies of QFT-IT sensitivity [7], [14], [16], [20]–[22]. Nevertheless, studies carried out in similar populations, Tsiouris et al [14] and Raby et al [16], reported sensitivities of 74% and 76% respectively, which is comparable to our findings. In HIV-negative patients we found a sensitivity of 81% which is fully comparable to previous studies in high income populations [7], [20], [21], [23].

We have shown that test performance is impaired in HIV-positive patients, as has also been suggested by previous studies: While Rangaka et al [10] found no influence of HIV-status on test performance for diagnosing LTBI, other studies have shown that HIV-status and immune deficiency does in fact affect test performance for the diagnosis of both LTBI and active TB infection [5], [7], [10]–[16]. Raby et al [16] reported a decrease in sensitivity of 21 percentage points in HIV-positive compared to HIV-negative patients when including indeterminate results and other studies of test performance in HIV-positive patients have reported similar findings [18].

Since the cut-off point of the test (0.35 IU/ml) is very low compared to the rather wide range of IFN-γ production (assay sensitivity up to 10 IU/ml), the absolute IFN-γ values might be difficult to interpret compared to the results deducted from the algorithm, which merely considers whether a data point lies above or below the cut-off value. They may, however, be useful in illustrating some inherent properties of the test. Our data demonstrate a lower median antigen dependent IFN-γ production in HIV-positive compared to HIV-negative patients. This difference was not observed when comparing HIV-positive and HIV-negative patients within the isolated result groups (positive, negative and indeterminate respectively) and we found no difference in median IFN-γ production between patients with CD4 cell count above and below 300 cells/µl. This might however be due to lack of power. Although one of the advantages of the QFT-IT often emphasized is the dichotomous test outcome, the cut-off is still a matter of debate and research [24], [25]. In our study, two patients with a negative and one patient with an indeterminate QFT-IT result had an antigen dependent IFN-γ response very close to the cut-off value of 0.35 IU/ml (0.32, 0.34 and 0.34 IU/ml respectively; data not shown).

Similar to the findings of Raby et al [16], we have demonstrated a clear trend of decreasing sensitivity with decreasing CD4 cell count in HIV-positive patients. Other studies have also found an association between low CD4 cell count and low QFT-IT sensitivity and/or high levels of indeterminate results in HIV-positive patients [12]–[14], [18], [19], [26], while other studies do not reproduce this finding [5], [10], [27]. The division of CD4 cell count into ranges of hundreds was chosen due to the straightforward clinical interpretation, but this also skews the data somewhat as the number of patients in each group is far from equal. Nevertheless, the trend was also found when dividing CD4 cell count groups into percentiles with equal numbers of patients in each group. In our study, the observed trend was mediated by an increasing number of indeterminate results with low CD4 cell count, since, when excluding indeterminate result, the trend could not be reproduced. Also, when excluding indeterminate result, we found no difference in sensitivity between HIV-negative and HIV–positive patients and no difference in median level of antigen dependent IFN-γ production. Nevertheless, a low CD4 cell count was not associated with indeterminate results in HIV-negative patients which might suggest that anergy of HIV-infected cells in HIV-positive patients makes adequate cytokine production more dependent on absolute cell numbers.

Although we found a larger proportion of indeterminate results (23/161 = 14%) than the majority of previously published studies, the proportion is similar to the proportion reported from studies from similar populations: Tsiouris et al [14] found 23 out of 154 (15%) results to be indeterminate while Raby et al [16] found 16 out of 112 (14%) results to be indeterminate. Similar to the findings of these studies, the majority of the indeterminate results was found in HIV-positive patients and could be explained by a low CD4 cell count.

The PHA positive control is a marker of immune mediated anergy and serves to eliminate a large proportion of otherwise false negative test results by classifying them instead as “indeterminate”. From a clinician's point of view, the sensitivity should address the risk that the test is false negative, i.e. the sensitivity for a valid QFT-IT response. This is expressed by the sensitivity when excluding indeterminate results. In our study the sensitivity when excluding indeterminate results was markedly better (86%), especially in HIV-positive patients: sensitivity increased from 65 to 83% in HIV-positive and from 81 to 88% in HIV-negative patients with no difference in sensitivity between the two groups (p = 0.45). Similar improvements in sensitivity when excluding indeterminate results have been found for HIV-positive patients in other studies [14], [16]. Although reporting an improvement from 63 to 78%, Raby et al [16] still found a lower sensitivity in HIV-positive than HIV-negative patients after correcting for indeterminate results (chi-square test, p = 0.01; calculated by authors from crude data in article). The study also found a significant association between a negative QFT-IT result and low CD4 cell count which may explain this finding. However, this particular association was not reproduced by our data.

All indeterminate results were due to an inadequate IFN-γ production in response to both the PHA positive control and the *M.tb.*-specific antigens. While the majority of these results were related to a low CD4 cell count as illustrated by the trend in Figure 3, indeterminate results also occurred in 7 out of 73 (10%) HIV-negative patients with a CD4 cell count above 300 cells/µl (data not shown). Other factors yet unidentified may account for these results. By multivariate analysis we found that male patients had a greater risk compared to females of having an indeterminate QFT-IT result (table 3). This is in contrast to the findings of Chee et al. [23] and the findings may reflect behavioural or social differences between the sexes rather than physiological properties. Also, we found that patients aged 33 or older were more likely to have an indeterminate results, but this was by univariate analysis only.

Even when excluding the indeterminate results, the QFT-IT still missed 19 (12%) of patients with culture confirmed TB disease. Previous studies have described a connection between a false negative QFT-IT result and age above 60 years, female sex and CD4 cell count [10], [16], [23]. Our data did not reproduce these findings. Also, a connection between false negative results and advanced cavitary disease has been suggested, although data on the subject has not been consistent [22], [28]–[33]. Our results did not support this suggestion as patients with negative or indeterminate QFT-IT results did not have higher bacterial loads by sputum microscopy, which has been shown to be associated with cavitary disease [34], [35]. An alternative explanation for the false negative results might be antigen specific anergy of sensitised T-cells or homing of the T-cells to the site of infection; phenomenons which have previously been described in connection with both PPD antigen tests and a similar Interferon Gamma Release Assay (the T-SPOT.*TB*) [36], [37].

As previously mentioned, potential uses of the IGRAs in TB- and HIV-endemic settings include exclusion of TB disease in patients suspected of TB disease as well as screening of risk populations, e.g. HIV-positive persons, for LTBI allowing for targeted preventive therapy [3]. Immunoassays do indeed seem to be promising candidates for replacement of the TST in the diagnosis of LTBI, however we and others have shown that to some extent test performance is impaired in HIV-infected patients with low CD4 cell counts. Furthermore, studies in these regions have found that up to 50% of the adult population is IGRA positive [10], but not necessarily suffering from active TB, possibly compromising specificity for diagnosing and excluding active TB infection. This issue was not the focus of our study but is currently being addressed in a community study in the same area. A recent study suggests that IGRAs may be useful in children where the risk of exposure due to young age is much lower [38].

Measures might be taken in order to improve the performance of immunoassays: re-evaluation of the recommended cut-off value might prove beneficial [24], [25] as might the exploration of alternative biomarkers for TB diagnosis [39]. Considering the financial and technical requirements of the test and the reduced specificity and sensitivity for active TB infection in areas where LTBI and HIV-infection is prevalent, cost-effectiveness is most likely low in the areas with the highest burden of TB infection. Nevertheless immunoassays have plenty of potential for improvement and further studies are needed to investigate how to optimise IGRA performance in TB- and HIV-endemic populations.

## Materials and Methods

### Setting, patient recruitment and eligibility

The study was conducted in Mwanza City, the second largest city in Tanzania. The TB incidence and HIV prevalence among the adult population (15–49 years) was estimated to be 342 per 100 000 and 6.5% respectively (2005) [40]. HIV prevalence in active TB cases was estimated to be 29% [40], however a recent study conducted in Mwanza, Tanzania found that 44% of the sputum smear positive patients were HIV-infected [41].

The study was conducted from April through December 2006 as a sub-study within the framework of two on-going randomised nutritional supplementation trials in TB patients. In Tanzania all patients presenting to a public health facility with TB-suspicious disease are referred to the National Tuberculosis and Leprosy Programme of Tanzania (NTLP) for diagnosis and treatment free of charge. PTB-diagnosis is based primarily on sputum smear microscopy or clinical criteria as by WHO recommendations and definitions [42]. PTB patients were recruited in four facilities (two hospitals and two health centers) in Mwanza City through the NTLP. Apart from inclusion in the NTLP, inclusion criteria were: consent to donate blood for the relevant tests as well as claiming it likely that they would remain in the study are during the entire study period. Exclusion criteria were: age below 15 years, pregnancy or lactation and/or presence of serious co-morbidity. Patients where no valid HIV result could be obtained were also excluded from the study. For this sub-study only patients with a positive sputum culture for *M.tb.* were included.

### Study assessments and measurements

#### i) Sputum microscopy and culture

Routine sputum microscopy (Ziehl-Neelsen sputum smear staining technique) for acid fast bacilli (AFB) was performed as part of the NTLP of Tanzania. Smears were graded according to the IUATLD standard [43] as follows: 0 (no AFB on smear), 1–9 (exact number of AFB per 100 fields), 1+ (10–99 AFB per 100 fields), 2+ (1–10 AFB per field) and 3+ (more than 10 AFB per field). Sputum smear grade was based on the smear with the largest amount of AFB found. Upon inclusion, an additional sputum sample was collected for culture for *M.tb.* performed on egg-based Lowenstein-Jensen solid media at the Zonal Tuberculosis Reference Laboratory at Bugando Medical Centre, Mwanza.

#### ii) HIV testing, lymphocyte and CD4 counts, clinical parameters

HIV testing was performed in duplicates using rapid tests Determine HIV 1/2 (Inverness Medical Innovations, Inc., Delaware, U.S.A.) and Capillus HIV-1/HIV-2 (Trinity Biotech Plc., Wicklow Ireland). Any indeterminate results were repeated and confirmed using ELISA – Organon Uniform II (Organon Teknia, the Netherlands). Lymphocyte count was obtained by direct microscopy of Leishman-stained blood smears. CD4 cell counts were obtained using the Partec FACS (Partec GmbH,. Münster, Germany). Body mass index (BMI) was calculated as weight (kg) divided by height (m) squared.

#### iii) QuantiFERON-TB® Gold In-Tube (QFT-IT) test

Blood was collected from study participants in heparinized tubes (Lithium heparinized plasmacontainers 4 ml, BD Vacutainer®, BD Denmark, Brøndby, DK) in each of the four main study centres and transported to the study laboratory within 8 hours. One ml of blood was transferred to each of the 3 QFT-IT test blood collection tubes pre-coated with the TB-specific antigens, a phytohemagglutinin (PHA) positive control (Mitogen) and a negative (Nil) control tube respectively. The samples were then shaken in order to ensure proper mixing of blood with contents and incubated upright at temperature 37°C for 20–24 hours. They were centrifuged at 3,000×*g* for 10 minutes and plasma was harvested and collected in Nunc® cryotubes (Nunc, Roskilde, Denmark) and stored frozen at −80°C. Plasma samples were transported on dry ice from Tanzania to the Department of Infectious Diseases at Copenhagen University Hospital, Hvidovre, Denmark, where ELISA testing was performed. Samples were tested in random order and laboratory personnel were blinded with regard to sputum-, culture- and HIV-status of the patients. Samples from up to 28 patients were ELISA tested at a time and the ELISA was performed in accordance with the instructions of the manufacturer (www.cellestis.com). ELISA readings were transferred to the QFT-Gold v.2.50 Software giving final QFT-IT test results as calculated by manufacturer's recommendations. The QFT-IT result was graded as positive if the IFN-γ in plasma of the antigen-stimulated blood was >0.35 IU/ml after subtracting the cytokine concentration of unstimulated plasma and the stimulation index (IFN–γ concentration in plasma of antigen stimulated blood divided by IFN–γ concentration of unstimulated blood) was ≥1.25 regardless of the mitogen stimulated IFN–γ response. Responses were graded as negative if the antigen-specific response was <0.35 IU/ml and the mitogen stimulated IFN-γ response was ≥0.5 IU/ml. Responses were indeterminate if either the IFN-γ response in the unstimulated sample was ≥8 IU/ml regardless of the antigen-specific and the mitogen-stimulated responses or if both the antigen-specific response was <0.35 IU/ml and the mitogen-stimulated IFN-γ response was ≤0,5 IU/ml.

#### iv) Statistical analysis

Data were entered and analyzed using SAS 9.1 for Windows (SAS Institute Inc., Cary, NC, USA). Sensitivity estimates were calculated using only patients with a positive sputum culture for *M.tb*. Analyses were done two-sided, confidence intervals (CI) were 95% and a result was considered significant when the p-value was below 0.05. Continuous variables were tested for normality using the Shapiro-Wilk test and compared using the Student's t-test if normally distributed; otherwise the Mann-Whitney test was applied. The Chi-square test was applied for comparing categorical variables unless one of the categories had less than 20 observations in which case the Fisher's exact test was applied. Test for trend was done using the Cochrane-Armitage test and correlation calculations were done using the Spearman non-parametric method. Odds ratio (OR) analysis for risk factors was performed by both uni- and multivariate analysis.

### Ethical considerations

The permission to conduct the study was granted by the ethics committee of the National Institute for Medical Research (NIMR) in Tanzania and was approved by The Danish National Committee on Biomedical Research Ethics in Denmark (reference no. 2005-7041-57). All study participants gave informed consent and were free to withdraw from the study at any time. For patients less than 18 years of age permission and informed consent was collected from parent or legal guardian. Pre-HIV test counselling was offered before patients gave final consent to allow for HIV-testing. HIV-positive individuals were offered post-HIV test counselling and were referred to the local HIV care and treatment clinics with information on HIV test result and CD4 cell count.
